# Caldera collapse thresholds correlate with magma chamber dimensions

**DOI:** 10.1038/s41598-023-34411-5

**Published:** 2023-05-08

**Authors:** Nobuo Geshi, Isoji Miyagi, Genji Saito, Chris E. Conway

**Affiliations:** Research Institute of Earthquake and Volcano Geology, Geological Survey of Japan, AIST, National Institute of Advanced Industrial Science and Technology, AIST Site 7, 1-1-1 Higashi, Tsukuba, Ibaraki 305-8567 Japan

**Keywords:** Natural hazards, Petrology, Volcanology

## Abstract

Explosive caldera-forming eruptions eject voluminous magma during the gravitational collapse of the roof of the magma chamber. Caldera collapse is known to occur by rapid decompression of a magma chamber at shallow depth, however, the thresholds for magma chamber decompression that promotes caldera collapse have not been tested using examples from actual caldera-forming eruptions. Here, we investigated the processes of magma chamber decompression leading to caldera collapse using two natural examples from Aira and Kikai calderas in southwestern Japan. The analysis of water content in phenocryst glass embayments revealed that Aira experienced a large magmatic underpressure before the onset of caldera collapse, whereas caldera collapse occurred with a relatively small underpressure at Kikai. Our friction models for caldera faults show that the underpressure required for a magma chamber to collapse is proportional to the square of the depth to the magma chamber for calderas of the same horizontal size. This model explains why the relatively deep magma system of Aira required a larger underpressure for collapse when compared with the shallower magma chamber of Kikai. The distinct magma chamber underpressure thresholds can explain variations in the evolution of caldera-forming eruptions and the eruption sequences for catastrophic ignimbrites during caldera collapse.

## Introduction

Caldera-forming eruptions (CFE) are characterized by the gravitational collapse of the roof of a magma chamber by rapid extraction of magma from the chamber^1,2^. Collapse calderas occur in various tectonic environments on Earth such as subduction zones, hotspots, and rift zones^3^. Explosive CFE that eject several tens to hundreds of cubic kilometers of magma are capable of causing catastrophic devastation to natural and built environments around volcanoes, and can also produce abrupt global climate disturbances due to the injection of voluminous volcanic ash and aerosols into the atmosphere^4–7^. Therefore, understanding the mechanisms of CFE is an important research issue for a wide range of sciences beyond volcanology.

**Figure 1:**
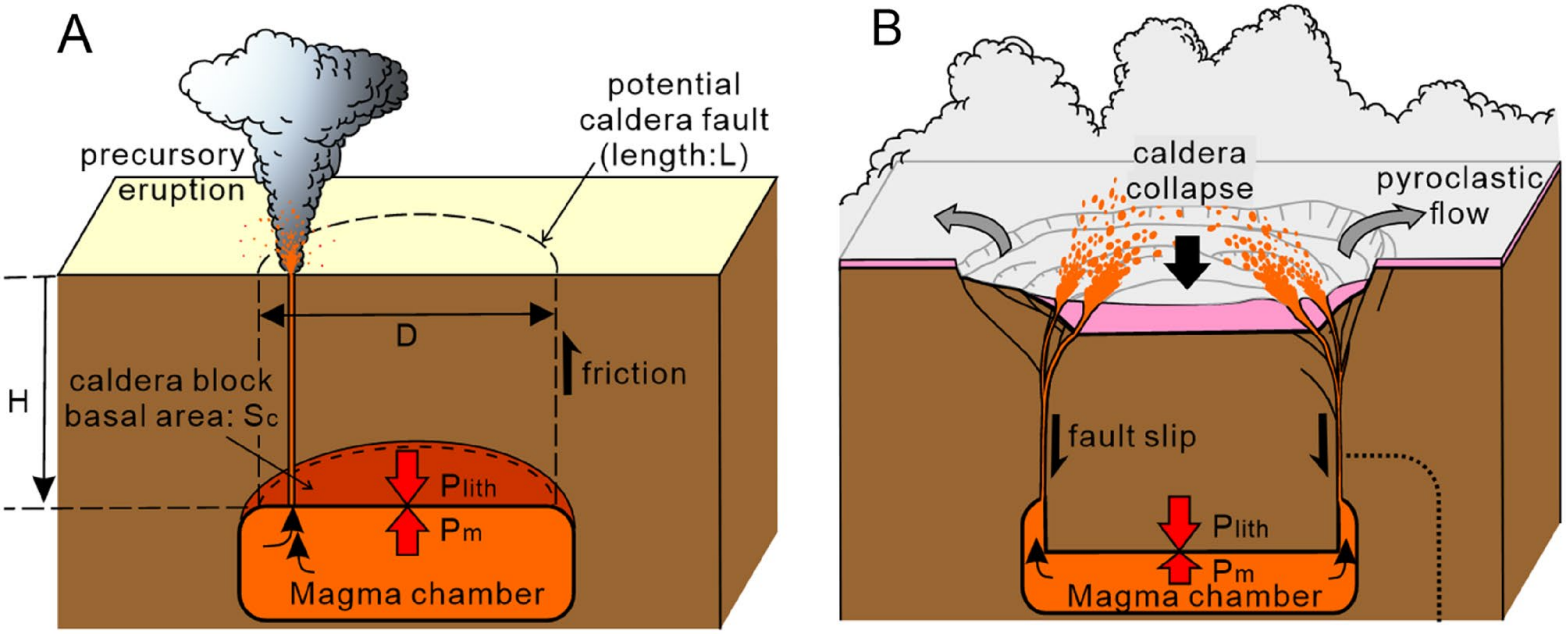
Schematic illustrations of caldera collapse. (**A**) Extraction of magma from magma chamber at depth H causes the decrease of magma pressure P_m_ from the lithostatic pressure at the magma chamber P_lith_. The difference between P_lith_ and P_m_ at the roof of the magma chamber drives the collapse of the caldera block. Friction on the potential caldera faults prevents the collapse of the caldera block. (**B**) When the differential pressure exceeds the caldera fault’s friction force, the caldera block collapses into the chamber. Collapse of roof rock into the magma chamber boosts the extraction of magma through the fractures and results in the eruption of a massive pyroclastic flow.

CFE are clearly distinct from other small eruptions that accompany magma chamber collapse by rapid magma extraction (Fig. 1^1,2,8^). Caldera collapse occurs when the downward force acting on the roof block of a magma chamber (i.e., the difference between magma chamber pressure and lithostatic pressure) exceeds the friction on the caldera fault (Fig. 1A^9–12^). Since the compression of the magma chamber by collapse of the caldera block can boost the rapid extraction of magmas from the magma chamber through ring fractures and result in the emplacement of massive pyroclastic flows (Fig. 1B), magma chamber decompression is the key process within the onset and evolution of CFE.

Various theoretical studies have been conducted on the pressure evolution of magma chambers leading to caldera collapse^9–12^. These models predict variations in the pressure evolution of magma chambers for caldera collapse, from “overpressure caldera” leading to CFE with excess pressure to “underpressure caldera” leading to collapse with sufficient decompression by the extraction of magma. Large variations in erupted magma volumes prior to caldera collapse among CFE indicate that the magnitude of magma chamber decompression for collapse is also widely variable among “underpressure calderas”^11^. The sequence for CFE includes cases where voluminous magma erupts as a single pulse of pyroclastic flow^13^ and cases where magma erupts as multiple pulses^14^ that are separated by decreases in eruption intensity or complete cessations, suggesting a diversity of magma chamber decompression processes that control the caldera collapse. Though some conceptual models of the development of pressure in magma chambers linked to the variation of CFE have been proposed^9,15^, these models have not been examined for natural examples due to the difficulty of estimating magma chamber pressures from natural volcanic products. Uncertainties regarding magma chamber decompression processes hinder our ability to understand and model CFE.

**Figure 2:**
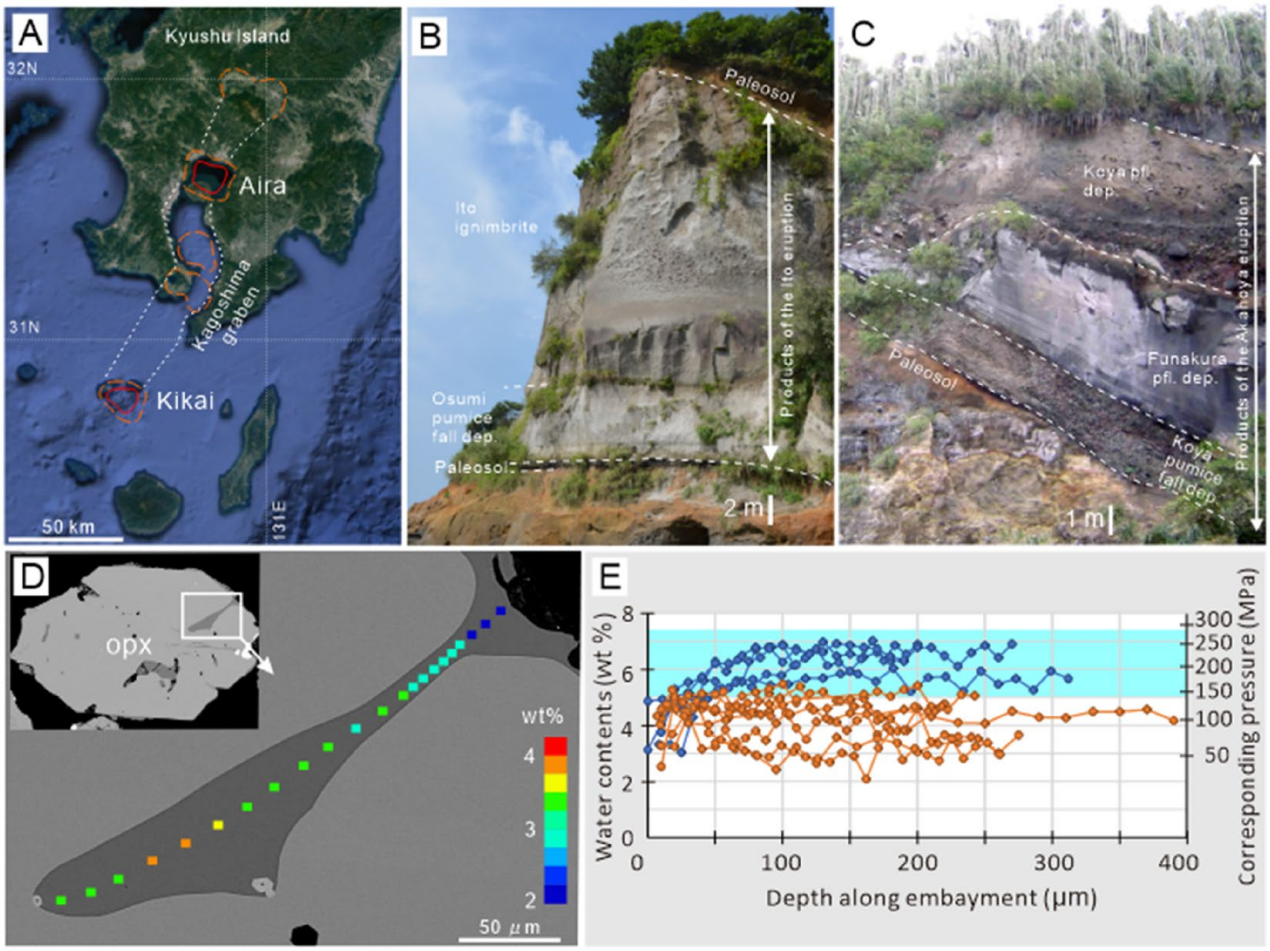
(**A**) Location of Aira and Kikai calderas in Kagoshima Graben. Structural margins (red) and topographic margins (orange) of each caldera are shown. Map image is a Google Earth image (Imagery date: 2015/12/14. Google Data SIO, NOAA, U.S. Navy, NGA, GEBCO Landsat/Copernicus). (**B**) Deposit of the Ito eruption of Aira caldera exposed at ~ 45 km southeast of the source. (**C**) Deposit of the Akahoya eruption of Kikai caldera exposed at the northern topographic rim of the caldera. (**D**) Backscattered electron image of a glass embayment hosted in an orthopyroxene phenocryst from the Akahoya eruption. Color of each square shows the water content at each location. (**E**) Profiles of the water content along glass embayments from Aira caldera eruptive products. Blue and orange dots show the profiles obtained from the lower part and upper part of the initial Plinian pumice fall deposit, respectively. Blue band shows the ~ 90% range of the water contents in the glass inclusions of Aira, indicative of the magma storage pressure condition.

Here, we present a decompression model for magma chambers during CFE, based on two contrasting VEI 7 class eruptions of the Aira and Kikai calderas in Kagoshima, southwestern Japan (Fig. 2A^13,16–19^). We traced the evolution of magma chamber decompression during these CFE, using the sequential changes of water contents in magma recorded by glass inclusion and embayments in the phenocrysts in the eruptive products. The distinct magma chamber decompression processes for these case studies indicate that the structure of the caldera faults controls the decompression and collapse processes and the sequence of CFE.

### Caldera-forming eruptions of Aira and Kikai

Aira caldera is the source caldera of a VEI 7 ignimbrite eruption (called Ito eruption) at around ~ 30,000 years ago^13,20^. The Ito eruption ejected a total of ~ 400 km^3^ dense-rock equivalent (DRE) of high-silica rhyolite magmas as a Plinian pumice fall deposit (Osumi pumice fall deposit; ~ 40 km^3^ in DRE^21^, corresponding to ~ 10% of the total erupted magma), transitional ignimbrite (Tsumaya ignimbrite; ~ 10 km^3^^20^), and main ignimbrite (Ito ignimbrite and its co-ignimbrite ash Aira-Tn ash fall deposit) in sequential order^13^. The lack of clear evidence of a time gap during the ignimbrite eruption, suggests that all units of the AT eruption were emplaced continuously within a short period. Based on the ratio of the total volume of the erupted magma and the discharge rate, the initial Plinian eruption toward the onset of the caldera collapse lasted a couple of days^21^.

Kikai caldera produced a VEI 7 ignimbrite eruption (Akahoya eruption) at around 7300 years ago^18^. The Akahoya eruption ejected more than 100 km^3^ DRE^22^ of rhyodacite magmas as Plinian pumice fall (named Koya pumice fall deposit^23^; more than 7 km^3^ DRE^22^ corresponding to ~ 7% of the total erupted magma), transitional ignimbrite (Funakura pyroclastic flow), and main ignimbrite (Koya pyroclastic flow and its co-ignimbrite ash Akahoya ash fall deposit^17,18^). A significant time gap is recognized between the Funakura pyroclastic flow in the early stages of the eruption and the Koya pyroclastic flow in the later stage^24^. The collapse caldera of the Akahoya eruption overprinted the previous caldera which was formed by another VEI 7 class eruption (Tozurahara eruption) around 95,000 years ago^25^.

Aira and Kikai calderas are considered to be “decompression calderas^15^”, which were formed by significant decompression of their magma chambers, since the collapse of both calderas was preceded by the explosive eruptions of several tens of km^3^ of magmas.

### Magma chamber decompression

**Figure 3:**
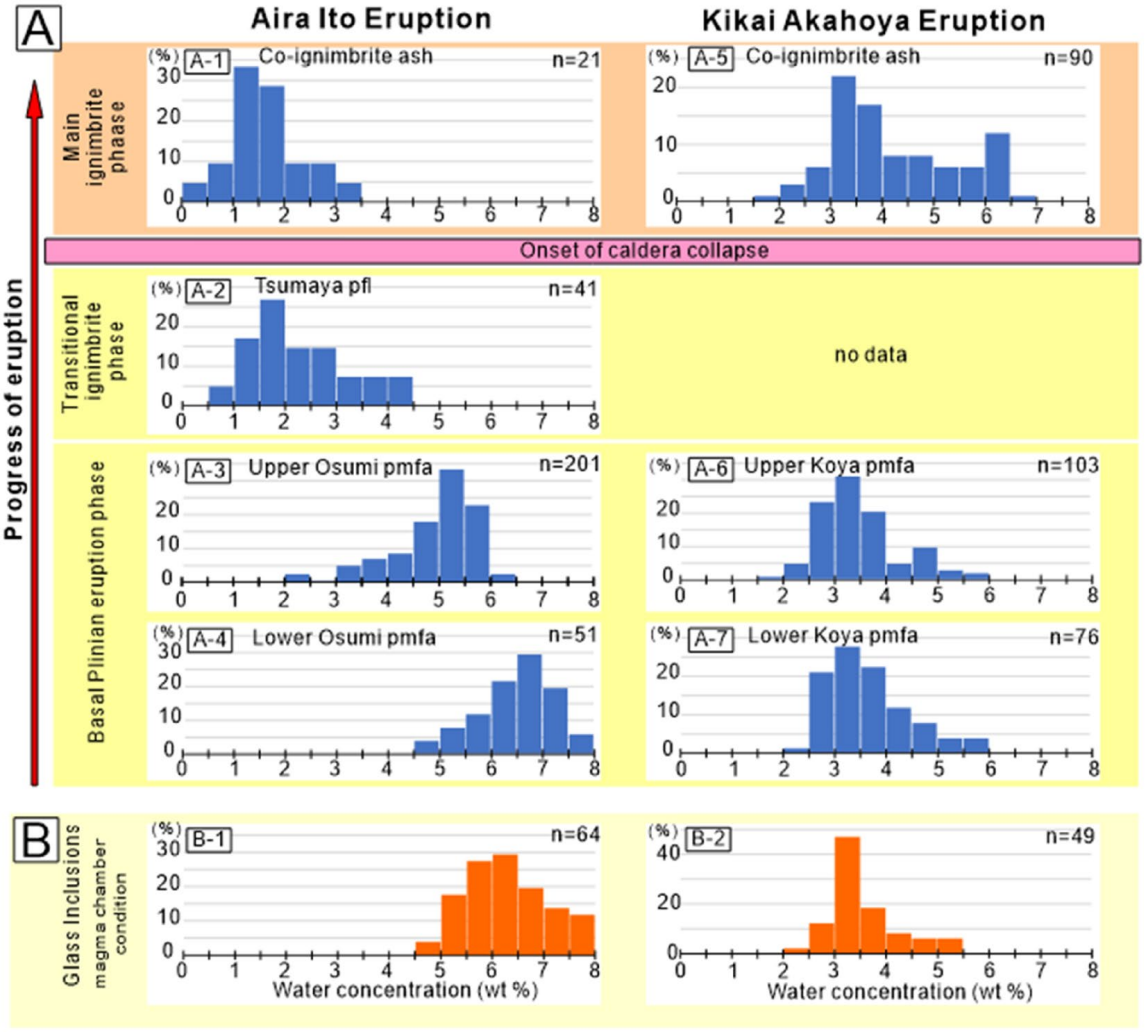
(**A**) Water content in glass embayments from the products of the Ito eruption of Aira and the Akahoya eruption of Kikai. Data of the Ito eruption is after^20^. Vertical scale is the frequency (unit: percentage). Water concentration data from deeper than ~ 100 μm from the entrance of embayments were used. Number of data is shown in upper-right of each panel. Average of two to four spot measurements in each melt embayment is used for one data. Horizontal axis shows the water content in weight per cent. (**B**) water contents in glass inclusions from the products of the Ito eruption of Aira (B-1) and the Akahoya eruption of Kikai (B-2).

We investigated the change of water contents in the deeper parts of glass embayments along the stratigraphic sequence for deposits from CFE of Aira and Kikai calderas (Fig. 2B,C). The water concentrations in the deeper parts of glass embayments, which were not affected by decompressional dehydration during rapid conduit ascent^20^, were used as indicators of the pressure conditions in the magma chambers (Fig. 2D^20^). Aira and Kikai show contrasting water concentration variations in glass embayments during their CFE, suggesting their magma chambers experienced different pressure evolution pathways. Aira shows a systematic decrease of the water content in glass embayments along the stratigraphy of the products of the Ito eruption, indicating that decompressional dehydration of the magma chamber occurred in the lead-up to caldera collapse^20^. The water concentrations in the glass embayments stay at ~ 5–6 wt% in the lower half of the Osumi pumice fall deposit, then start to decrease to 3.5–5.5 wt% at the top of the pumice fall deposit (Fig. 2E), and finally decrease to 2–4 wt% for the Tsumaya pyroclastic flow deposit which erupted just before the caldera collapse (Fig. 3A). This decrease of water contents corresponds to decompression from 136–192 MPa to 27–90 MPa assuming the saturation of water in melt^26^. In contrast, the water concentrations in the glass embayments of the Akahoya eruption of Kikai caldera show no clear change throughout the eruption (Fig. 3A), suggesting only a minor decompression of the magma chamber during the eruption. The water concentrations in the plateau part of the glass embayment of the Koya pumice fall deposit range between 2.5 and 4.0 wt%. This water contents corresponds to the saturation concentration at 39–90 MPa.

These two calderas have contrasting magma chamber depths. The depth of the magma chambers, indicated by the water content of the glass inclusions in phenocrysts (Fig. 3B), is estimated to be about 5 km for Aira and 3 km for Kikai^20,27,28^. The water concentrations in the glass inclusions of the Ito eruption of Aira (5.0–7.0 wt%) correspond to the saturation pressure of water at 136–257 MPa, which correspond to the lithostatic pressure at 5.1–9.7 km depth, with the assumption that the density of the host rock is 2700 kg m^−3^. The water concentrations in the glass inclusions in the products of the Akahoya eruption of Kikai (2.5–4.0 wt%) correspond to the saturation pressure of water at 39–90 MPa, which corresponds to the lithostatic pressure at 2.0–3.4 km depth. Assuming that the range of the water content in each caldera shows the variation of the crystallization depth of phenocrysts, the smallest value of the depth obtained from the glass inclusions represents the roof depth of the magma chamber.

## Discussion

### Magma chamber depth and underpressure

To explain the relationship between the variation of the roof depth and the pressure evolution of the magma chamber during CFE, we examined the relationship between the depth and horizontal size of magma chambers, and the magnitude of underpressure for triggering caldera collapse using a piston-cylinder caldera model (Fig. 1A).

The stress acting on the potential caldera faults in the roof rock of the magma chamber increase as the decompression of the magma chamber by magma extraction progresses. Caldera subsidence starts when the driving force pulling the caldera block down into the magma chamber exceeds the frictional force acting on the caldera fault plane^11,12^. Therefore, the critical underpressure for caldera collapse *P*_*u*_ can be given as

1 $$\begin{array}{c}{P}_{u}=\frac{1}{2}\mu \rho g\frac{L}{{S}_{c}}{H}^{2}\end{array}$$

where *μ* is the static friction coefficient of the fault, *ρ* is the density of the host rock, *g* is the gravitational acceleration, S_c_ is the basal area of the caldera block, *L* is the perimeter length of the caldera block, *H* is the depth to the magma chamber. Here, we assume that the density of the host rock *ρ* and the friction coefficient of the host rock *μ* are 2700 kg m^−3^ and 0.6, respectively, based on the typical value of the granitic rock and consolidated sedimentary rocks that host these calderas. The gravitational acceleration g is assumed to be 9.8 ms^−2^.

Assuming a cylindrical caldera block surrounded by vertical caldera faults for Aira and Kikai, Eq. (1) shows that Aira (*S*_*c*_ = 200 km^2^, *L* = 50 km, *H* = 5.3 km) requires an underpressure of ~ 61 MPa for collapse, whereas Kikai (*S*_*c*_ = 200 km^2^, *L* = 55 km, *H* = 3 km) can collapse with ~ 18 MPa of underpressure. Since the sizes of Aira and Kikai are similar, this difference in underpressure for caldera collapse is mainly caused by the difference in the depth to the magma chambers in each caldera system. A large underpressure is required for caldera collapse for Aira with its deeper magma chamber, whereas a relatively small underpressure can trigger caldera collapse at Kikai with its shallower magma chamber, as recorded in the water contents in the glass embayments from eruptive products of these calderas.

Underpressure for caldera collapse is also affected by the friction on the caldera fault. Repeated slip and hydrothermal alteration along the fault may decrease the friction coefficient *μ* on a caldera fault, thus requiring a smaller underpressure for caldera collapse. Reactivation of an existing caldera fault is therefore expected to permit collapse with smaller underpressure than a collapse caldera without a pre-existing structure. The Aira caldera formed in a position where no previous caldera structure existed. In contrast, the Akahoya eruption was, insofar as is known, the second caldera-forming event of the Kikai caldera, which may have subsided by reactivation of the existing caldera fault with smaller friction. The presence of the previous caldera structure, in addition to the shallower magma chamber depth, may have contributed to caldera collapse during the Akahoya eruption with smaller underpressure.

### En-mass and multiple collapse

The difference in underpressure thresholds for the two case studies shown here has implications for the mechanism of multiple pyroclastic flows interspersed by short pauses often seen in CFE. As shown by Eq. (1), a caldera fault can be activated by a lower underpressure in a magma chamber where the ratio $L/{S}_{c}$ and the friction on the fault surface are small, as in the case of Kikai. For these calderas, subsidence commences in the early stage of the eruption with small magma chamber underpressure. However, the subsidence of a caldera block by a small driving force can also be temporally locked by weak sticking on the caldera fault. Then, the caldera collapse will resume by failure of the minor sticking as the underpressure is recovered. This process can form multiple pyroclastic flows separated by a time break or periods of weaker eruptive activity. In the case of Kikai, a significant time gap is recognized between the Funakura pyroclastic flow in the early stages of the eruption and the Koya pyroclastic flow in the later stage^23,29^. Moreover, the Koya pyroclastic flow deposit also consists of several flow-units indicative of a pulse-like ignimbrite eruption. The Oruanui eruption of Taupō volcano, Aso-4 eruption of Aso volcano are another such cases for which a large ignimbrite was produced by multiple pulses separated by time breaks^14,30^. The shallow depth to the high-silica magma chamber (H ~ 3 km for Aso^30^ and H ~ 3.5 km for Taupō^31^) compared with the large caldera size for the Oruanui eruption of Taupō may have allowed caldera subsidence with a small underpressure, resulting in several breaks in the caldera collapse sequence.

**Figure 4:**
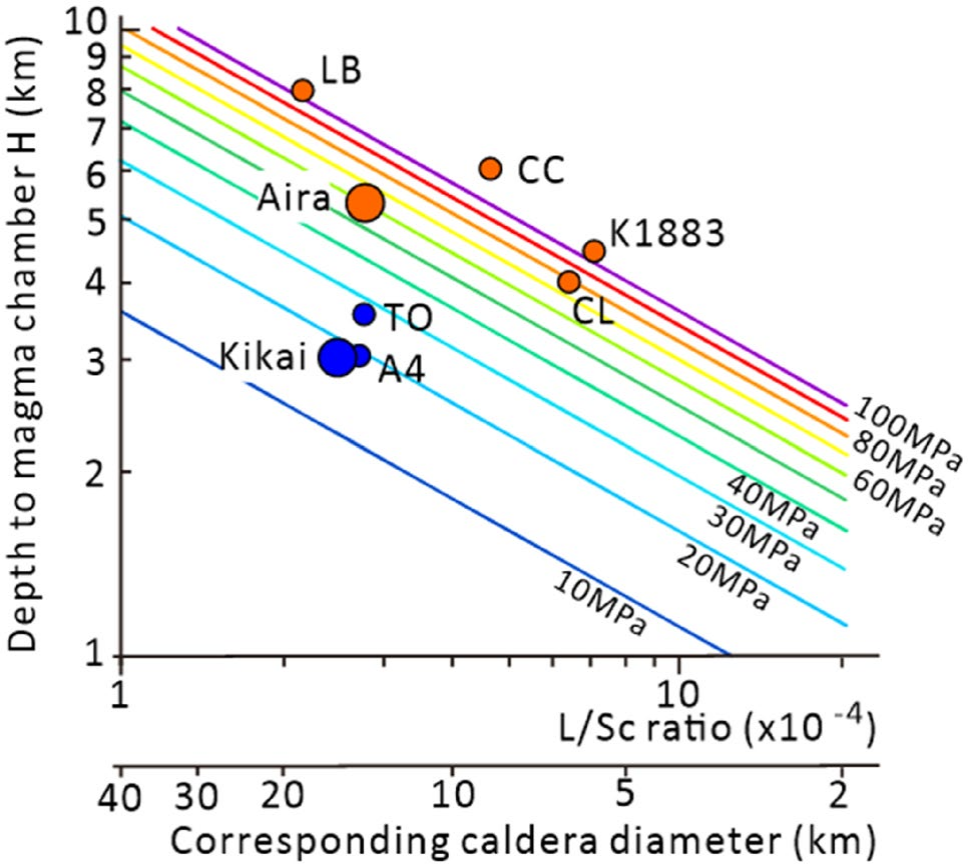
Critical underpressure in magma chamber for caldera collapse as the function of the depth to magma chamber roof and the L/S_c_ ratio of caldera fault based on Eq. (1). "Corresponding caldera diameter" at bottom axis is the caldera diameter corresponding to the L/Sc ratio, assuming a circular caldera shape. Large circles show the calculated decompression value of Aira and Kikai calderas. Small circles show the fault L/S_c_ and depth to the magma chamber of representative three calderas. TO: Oruanui eruption of the Taupō^14,31^, A4: Aso-4 eruption of Aso^30^, CC: Campanian ignimbrite eruption of Campi Flegrei^32,34^. LB: Bishop Tuff eruption of Long Valley^35^, CL: Crater Lake^36^, K1883: Krakatau 1883 eruption of Krakatau^37^. CFE of single major ignimbrite pulse are shown by orange color, and CFE with multiple ignimbrite pulse separated by time break are shown by blue.

A large underpressure in the magma chamber is required to trigger the collapse for calderas with larger $L/{S}_{c}$ ratio and higher friction on the fault surface, as for the case of Aira. Once subsidence of the caldera block is initiated, it is accelerated by the large driving force and continues until the pressure in the magma chamber recovers to the lithostatic pressure. Maturation of the fault plane as slip progresses and the fault is lubricated by the intrusion of magma and hydrothermal fluid may also promote a reduction of friction and an acceleration of the caldera subsidence. Continuous subsidence of a caldera block causes the continuous eruption of a large pyroclastic flow without significant time breaks. At Aira, the eruption and emplacement of the Ito ignimbrite without clear flow units reflects continuous caldera block subsidence driven by a large underpressure in the magma chamber. The Campanian Ignimbrite eruption of Campi Flegrei and the Bishop Tuff eruption of Long Valley are typical cases of CFE without any significant time breaks^32,33^. Relatively deep magma chambers at H ~ 6 km for Campi Flegrei^34^ and at H ~ 8 km^35^ for Long Valley compared with the horizontal size of the calderas may have required a large underpressure in the magma chamber at the onset of the caldera subsidence, as in the case of Aira. Larger underpressure in magma chamber is expected for the triggering of a collapse of smaller collapse caldera such as Crater Lake^36^ and Krakatau 1883^37^ due to their higher Sc/L ratio (Fig. 4). The large underpressure in these cases promoted continuous caldera block subsidence and production of a single-pulse ignimbrite ejection without any time gaps.

The comparison of the Aira and Kikai calderas reveals that caldera structure architecture and hysteresis can account for variations in the development of underpressure in magma chambers during CFE. Further integrated analyses of the horizontal scale of caldera faults, depth of magma chambers, and magma decompression pathways will advance our understanding of the timing and sequence of large-scale pyroclastic flow eruptions that are controlled by the underpressure of magma chambers. Caldera faults in nature are multiple and incline inward and/or outward, though we assume here a single and vertical caldera fault for simplicity of the model. Integrated models that more closely resemble actual caldera faults will provide a better understanding of CFE.

## Conclusions

Comparison of the caldera-forming eruptions of the Aira caldera and Kikai caldera shows the contrasting decompression process for each caldera. Aira experienced large underpressure in the magma chamber toward the onset of the caldera collapse, whereas Kikai experienced slight underpressure through the caldera forming eruption. A piston cylinder model of caldera collapse shows that the underpressure required for a magma chamber to collapse is proportional to the square of the depth to the magma chamber for calderas of the same horizontal size. This model can explain the larger decompression in the magma chamber is required for the Aira caldera with deeper magma chamber, whereas the slight decompression for the Kikai caldera with shallower magma chamber. Our results show that the size and depth of the magma chamber are critical parameters to the forecasting of the occurrence of catastrophic caldera-forming eruption, as the magnitude of the underpressure in the magma chamber may control the pattern of collapse and the eruption sequence of ignimbrite.

## Methods

### Sampling

#### Ito eruption of Aira caldera

Sequential samples of the Osumi pumice fall deposit of the Ito eruption of the Aira caldera were collected from three outcrops at Shinjo-Fumoto ~ 26 km southeast of the caldera center, Onohara at ~ 19 km southeast, and Futagawa ~ 12 km east of the caldera center. At Shinjo-Fumoto, the Osumi pumice fall deposit with approximately 5 m in thickness is exposed. The pumice clasts were collected from six different levels of the deposit at the outcrop. The uppermost part of the Osumi pumice fall deposit is interfingered with the overlying Tarumizu pyroclastic flow at Shinjo-Fumoto, thus, the Osumi pumice fall deposit may have been thermally affected by the overlying pyroclastic flow. To counter any potential effects, we also examined the uppermost part of the Osumi pumice fall deposit at the outcrops of Onohara and Futagawa, where the Osumi pumice fall deposit is not covered by the pyroclastic flow. The Osumi pumice fall deposit at Shinjo-Fumoto, Onohara and Futagawa are approximately 5 m, 12 m and 4 m in thickness, respectively. The top of the Osumi pumice fall deposit at both of the latter outcrops is directly covered by the fallout tephra deposit of the post-caldera Sakurajima. The pumice clasts were collected from the top part of the Osumi pumice fall deposit.

Tsumaya pyroclastic flow deposits of the Ito eruption were sampled from two outcrops at ~ 16.5 km northeast of the caldera center (Kokubu-Daimyoji, Kirishima City) and ~ 11 km northeast of the caldera center (Near Shiroyama Park, Kirishima City). At Kokubu-Daimyoji, the > 20 m-thick Tsumaya pyroclastic flow deposit covers the ~ 1.8 m-thick Osumi pumice fall deposit. At Shiroyama Park, the thickness of the Tsumaya pyroclastic flow deposit is ~ 8 m. The Tsumaya pyroclastic flow deposit at both outcrops consists of accretionary lapilli-rich, non-welded ash flow deposits. The pumice clasts were collected from the basal part of the Tsumaya pumice flow deposit.

Since the glass inclusions in the Ito ignimbrite were crystallized and dehydrated due to post-depositional thermal effects, we used the co-ignimbrite ash-fall deposit that separated during the eruption for the samples representing the main ignimbrite phase. The co-ignimbrite ash fall deposit of the Ito ignimbrite was sampled from outcrops ~ 40 km east of the caldera center (Onomi, Shibushi City), where the co-ignimbrite ash deposit is ~ 3 m thick.

#### Akahoya eruption of Kikai Caldera

Sequential samples of the Koya pumice fall deposit and Akahoya ash fall deposit of the Akahoya eruption of the Kikai caldera were collected from two outcrops at ~ 52 km northeast of the caldera center (Izashiki, Minami-Osumi), and ~ 77 km northeast of the caldera center (Aira-Kamimyo, Kanoya City). At Izashiki, the ~ 65 cm-thick Koya pumice fall deposit is covered by the ~ 50 cm-thick Koya ignimbrite, and then the 45 cm-thick Akahoya ash fall deposit. Pumice clasts and accretionary lapilli up to 2 cm in diameter are concentrated at the base of the Akahoya ash fall deposit. The pumice clasts were collected from five different levels of the Koya pumice fall deposit, and the basal part of the Akahoya ash fall deposit. At Aira-Kamimyo, the Koya pumice fall deposit of 15 cm thick is covered by the 25 cm-thick Akahoya ash fall deposit. Funakura pyroclastic flow deposit is not found at either outcrop, as the distribution of the Funakura pyroclastic flow deposit is limited to the caldera rim. The pumice clasts were collected from basal and upper parts of the Koya pumice fall deposit, and the basal part of the Akahoya ash fall deposit. We used the Akahoya ash-fall deposit for the samples representing the main ignimbrite phase as most of the glass inclusions in the Koya pyroclastic flow deposit were crystallized due to the post-depositional thermal effect.

### Chemical composition and water content

The phenocryst minerals were separated from the crushed and sieved pumice samples to determine the water concentration in the glass inclusions and embayments. Quartz and orthopyroxene crystals were collected from the samples of the Ito eruptions. Orthopyroxene and clinopyroxene crystals were collected from the samples of the Kikai-Akahoya eruption. Collected phenocrysts were fixed in an epoxy resin and polished to expose the glass inclusions and embayments. The polished surfaces were coated by carbon for analysis.

The water concentrations of the glass embayment were determined by an energy-dispersive X-ray spectrometer (EDS), X-Max 20 of Oxford Instrumentals, on a scanning electron microscope (SEM) JEOL JSM6610LV at the Geological Survey of Japan, following the method described in Geshi et al.^20^. The beam current for the measurement was 1.000 nA and the acceleration voltage was 15 kV.

For the determination of the water content in the glass, we used the stoichiometric balance between the oxygen and cation elements in the analyzed area^20^. Elements with larger atomic numbers than oxygen were quantified using EDS by the INCA software of Oxford Instruments. Assuming that all elements form oxides in glass, the quantified oxygen was distributed according to the valence of each element. All iron in the glass was in the form of ferric oxide (Fe^3+^). The water content in the volcanic glass was calculated assuming the excess oxygen in the glass forms H_2_O. Details of the method is described in Geshi et al.^20,38^.

Most of the samples were affected by the hydration from the surface of the glass after the eruption. The water concentration data in the glass embayment deeper than ~ 100 μm from the entrance of the embayment were used for analysis to avoid the effect of post-eruption hydration. Averages of two to four measurements in an embayment were used for the representative value of the embayment.

### Conversion of the water contents to saturation pressure

We converted water concentrations in the glass inclusions to the saturation pressure in the magma chamber, using the water solubility in rhyolite melt^26^. The partial pressure of water in the magma is assumed to be equal to the total magmatic pressure, as the concentrations of CO_2_ and other volatile phase in the glass are negligible (less than 250 ppm for the Aira^28^ and 40 ppm for the Kikai^27^). Presence of bubbles in these glass inclusions suggests that the melt was saturated in volatiles when they were trapped in the magma chamber. Presence of pheno-bubbles in these pumices^39^ also supports the saturation of volatiles in the magma chamber. We assumed that the differences in water concentrations in a single sample indicate the differences in the depth of crystallization of the phenocrysts in the magma chamber and the lower limit of the water concentration in a sample is considered to indicate the pressure conditions at the top of the magma chamber.

### Underpressure in magma chamber for caldera subsidence

The subsidence of the caldera block is driven by the magmatic underpressure acting on the base of the caldera block (roof of the magma chamber). Therefore, the driving force of subsidence acting on the caldera block *D*_*f*_ is written as

2 $$\begin{array}{c}{D}_{f}={S}_{c}{P}_{u}\end{array}$$

where S_c_ is the basal area of the caldera block and *P*_*u*_ is the underpressure at the roof of the magma chamber. Assuming the cylindrical caldera block is surrounded by a vertical caldera fault, the basal area of the caldera block *S*_*c*_ is equal to the structural caldera floor.

Friction on the fault plane prevents the subsidence of the caldera block. Based on Coulomb's friction law, the friction force on the caldera fault is expressed as

3 $$\begin{array}{c}{F}_{f}={S}_{f}\mu N\end{array}$$

where *S*_*f*_ is the area of the fault plane of the caldera fault, μ is the static friction coefficient of the fault, and *N* is the vertical stress acting on the fault. Assuming a cylindrical fault, *S*_*f*_ is given by *LH*. Average vertical stress on the caldera fault is assumed as the lithostatic pressure at the depth H/2, assuming a linear increase of lithostatic pressure with depth. Based on these assumptions, Eq. (3) can be modified as

4 $$\begin{array}{c}{F}_{f}=\frac{1}{2}L\mu \rho g{H}^{2}\end{array}$$

where *ρ* is the density of the host rock, *g* is the gravity acceleration, *L* is the perimeter length of the caldera block, *H* is the depth to the magma chamber. The perimeter length of the caldera block *L* is the outer circumference of the structural caldera floor assuming a cylindrical caldera block.

Caldera subsidence starts when the driving force pulling the caldera block down into the magma chamber *D*_*f*_ exceeds the frictional force acting on the caldera fault plane *F*_*f*_. Thus, the critical underpressure for caldera subsidence can be given as

5 $$\begin{array}{c}{P}_{u}=\frac{1}{2}\mu \rho g\frac{L}{{S}_{c}}{H}^{2}\end{array}$$

Equation (5) shows that the underpressure for the trigger of caldera collapse correlates with the ratio *L*/*S*_*c*_ and square of *H*. The ratio L/S_c_ is determined geometrically from the shape of the caldera floor. Since the ratio L/S_c_ is 2/r for a circular caldera with radius r, Eq. (5) can be modified for a circular caldera as

6 $$\begin{array}{c}{P}_{u}=\mu \rho g \frac{{H}^{2}}{r}\end{array}$$

where r is the caldera radius. Unless the caldera is extremely elongated, *P*_*u*_ is inversely proportional to the caldera diameter and proportional to the square of the depth *H*.

The parameters used in this model are dependent on the geology of the host rock of the caldera. The upper crustal materials that host the Aira and Kikai calderas consist of crystalline sandstone and mudstone of the Paleogene Shimanto Group, and Neogene granitic rocks intruding into them. . As the density of these rocks can range between 2500 and 2800 kg m^−3^^40^, we use the density ρ = 2700 kg m^−3^ for the host rock of the caldera. The maximum friction coefficient of representative dry silicate rocks ranges between 0.6 and 0.8^41^, though the presence of phyllosilicate minerals and water on the fault plane dramatically decreases the friction coefficient. Here, we use 0.6 as the friction coefficient of the host rock. Equation (5) shows that the obtained P_u_ is proportional to the friction coefficient.

## Data Availability

The datasets generated and analyzed during this study are available in “Figshare” repository, 10.6084/m9.figshare.21680480.v1, 10.6084/m9.figshare.15146955.v1.
